# Bioinspired Mechano‐Sensitive Macroporous Ceramic Sponge for Logical Drug and Cell Delivery

**DOI:** 10.1002/advs.201600410

**Published:** 2017-04-27

**Authors:** Changlu Xu, Zhihao Wei, Huajian Gao, Yanjie Bai, Huiling Liu, Huilin Yang, Yuekun Lai, Lei Yang

**Affiliations:** ^1^ Orthopaedic InstituteDepartment of OrthopaedicsThe First Affiliated HospitalSoochow UniversitySuzhouJiangsu215006P. R. China; ^2^ School of EngineeringBrown UniversityProvidenceRI02912USA; ^3^ School of Public HealthMedical CollegeSoochow UniversitySuzhouJiangsu215123P. R. China; ^4^ National Engineering Laboratory for Modern SilkCollege of Textile and Clothing EngineeringSoochow UniversitySuzhouJiangsu215123P. R. China; ^5^ International Research Center for Translational Orthopaedics (IRCTO)Soochow UniversitySuzhouJiangsu215006P. R. China

**Keywords:** bioinspired material, cell delivery, logic delivery, mechanically active, porous ceramic

## Abstract

On‐demand, ultrahigh precision delivery of molecules and cells assisted by scaffold is a pivotal theme in the field of controlled release, but it remains extremely challenging for ceramic‐based macroporous scaffolds that are prevalently used in regenerative medicine. Sea sponges (Phylum Porifera), whose bodies possess hierarchical pores or channels and organic/inorganic composite structures, can delicately control water intake/circulation and therefore achieve high precision mass transportation of food, oxygen, and wastes. Inspired by leuconoid sponge, in this study, the authors design and fabricate a biomimetic macroporous ceramic composite sponge (CCS) for high precision logic delivery of molecules and cells regulated by mechanical stimulus. The CCS reveals unique on‐demand AND logic release behaviors in response to dual‐gates of moisture and pressure (or strain) and, more importantly, 1 cm^3^ volume of CCS achieves unprecedentedly delivery precision of ≈100 ng per cycle for hydrophobic or hydrophilic molecules and ≈1400 cells per cycle for fibroblasts, respectively.

On‐demand delivery of molecules and cells is a pivotal theme in the field of controlled release. While most on‐demand delivery systems developed so far have placed much emphasis on achieving the release of cargos at controllable rates or by various release gates, some recently reported systems have achieved ultrahigh precision of release amount at nanogram regime.^1^, ^2^, ^3^ For biomedical applications like those in bone tissue engineering, ultrahigh precision release is highly desirable for the delivery of therapeutic cargos (such as bone morphogenetic proteins, parathyroid hormone, miRNA, etc.), for which accurate administration is imperative and the consequence of overdose is serious. Although porous scaffolds are highly efficient carriers for loading and transportation of biological entities, such as therapeutic molecules and cells,^4^, ^5^, ^6^, ^7^ it is very challenging to achieve highly precise, on‐command delivery of molecules and cells by porous scaffolds since they usually rely on passive release mechanisms (molecular diffusion, scaffold degradation, etc.).^8^, ^9^, ^10^, ^11^ Recent advances in porous scaffolds for high‐precision controlled release focus on developing active delivery systems that, to a large extent, achieve the delivery of biological cargos in response to external stimuli, such as temperature, pH, and enzyme.^12^, ^13^, ^14^, ^15^, ^16^ Owing to their responsive abilities to external stimuli, the active release systems possess considerable flexibility and versatility on the kinetics and amount of release.

Since most of the aforementioned active delivery systems are designed to operate under static conditions, their efficacy has a high likelihood to be compromised if implanted in the mechanically dynamic environment of target tissues in vivo. For mechanically different scenarios, such as trabecular bone (compressive modulus *E*_c_ > 50 MPa), cartilage (*E*_c_ = 0.1–2 MPa), and cardiac tissue (*E*_c_ = 0.01–0.02 MPa),^17^ the ability of delivery systems actively responding to such mechanically dynamic environment is highly desirable. Starting from the exemplary work by Mooney and co‐workers, mechanoresponsive materials triggered by mechanical stimuli (such as compression, tension, and shear) have demonstrated promising capacity of delivering cargos in a controlled and active manner.^18^ Nevertheless, there are only a few studies of active porous systems relying on mechanical stimuli for delivery.

Due to soaring demands for drug or cell delivery in force‐related biological systems like musculoskeletal and circulation systems, the new strategy of mechano‐sensitive delivery systems, which harness mechanical energy from host tissues to deliver wanted molecules and cells, have emerged.^19^ Recent examples include Fe_3_O_4_/alginate composite scaffolds delivering cells and drugs in response to variable magnetic forces^2^ and bacterial mechano‐sensitive channels acting like a dual‐control gate by cell membrane tension and MscL charges to modulate the delivery of bioactive molecules into live cells.^20^ These strategies, however, are not applicable in achieving ultrahigh release precision in ceramic‐based porous scaffolds due to the high rigidity, limited biodegradability, and large interconnected pores of ceramic scaffolds. Furthermore, delicate on‐demand release behaviors (including gated release, repeated release, or logic release) have yet to be realized in porous ceramic scaffolds possessing macropores (e.g., pore size >500 µm) and high porosity (e.g., >80%).

Inspiration from sea sponges (Phylum Porifera), which delicately masters water circulation through their bodies for food, oxygen, and waste exchanges,^21^ provides novel insights into the design and construct of mechano‐active porous ceramic structures for ultrahigh precision delivery. Leuconoid sponges have the most complex body structures among all types of sea sponge, consisting of a hierarchical channel system where spherical choanocyte chambers are connected by incurrent canals and apopyles (water exiting pores).^22^ The choanocytes, which are unique collar cells with flagella lining the internal chamber, take in water or expel it while capturing tiny food particles (nutrients, bacterial cells, etc.) in the interstices between choanocytes (**Figure** **1**a).^23^ Besides cells, the sponge has a skeleton composed of mesohyl (mainly collagen) and are structurally reinforced by calcite or silica spicules and/or spongin fibers,^24^ resembling essentially a ceramic‐polymer composite. This resilient composite, together with the hierarchical porous structure, renders the highly efficient water flow system for accurate transportation and exchange of materials from the nanometer scale to tens of micrometer (e.g., food, nutrient, waste particles, and gas molecules).

**Figure 1:**
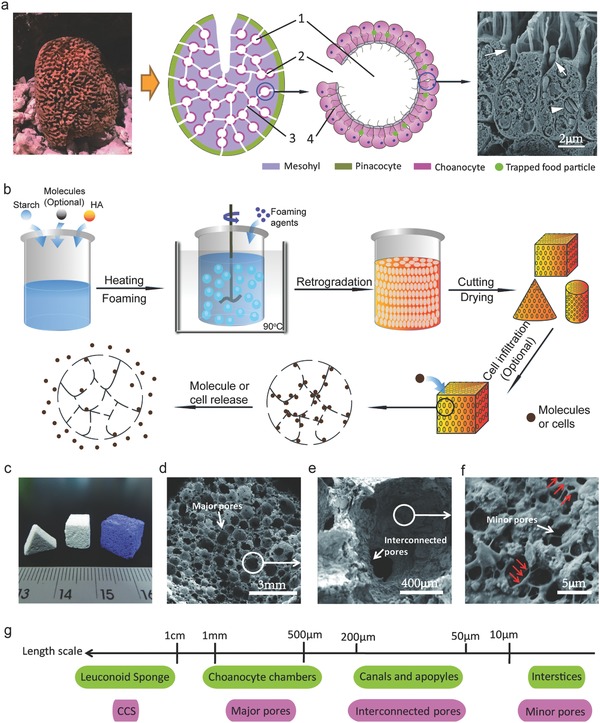
Leuconoid sponge‐inspired CCS with a hierarchically porous structure. a) Hierarchically porous structure of leuconoid sponge, 1‐choanocyte chamber, 2‐apopyle, 3‐canal and 4‐interstice. Arrow heads in the SEM image on the right indicate food particles and bacterial cells. b) Schematic diagram of the fabrication of CCS and molecule‐ or cell‐loaded CCS by a novel in situ foaming method. c) Photograph of CCS after being cut into different geometries (white) and BPB‐loaded CCS (blue). d) Three‐hierarchy porosity of CCS: spherical major pores in CCS, e) interconnected pores connecting adjacent major pores, and f) micrometer‐sized minor pores on the walls of major pores. g) Length scales of the hierarchical pores in leuconoid sponge and CCS. SEM image in (a) is reproduced with permission.^23^ Copyright 2006, The Marine Biological Laboratory.

Here, we report a mechanically regulated ultrahigh precision logic delivery system realized by bioinspired macroporous ceramic composite sponges (CCS). The CCS imitates the body plan and skeletal composition of leuconoid sponge to possess a three‐level hierarchy of porosity and high resilience, turning conventional rigid porous ceramics into mechano‐active sponges capable of programmable and repeatable deliveries of molecules and cells. We selected synthetic biomineral hydroxyapatite (HA) and natural cornstarch as CCS components and developed a novel foaming method to construct hierarchical structures, mimicking the leuconoid sponge. The resultant CCS is highly resilient and exhibits unique gate‐logic release behaviors with ultrahigh precision in response to mechanical stimuli. The mechanically regulated AND logic delivery system by a 1 cm^3^ CCS reaches an unprecedentedly delivery precision of ≈100 ng per cycle for hydrophobic or hydrophilic molecules and ≈1400 cells per cycle for fibroblasts. In addition, the compressive modulus of CCS changes significantly at different moisture contents, granting it flexibility to comply with the mechanical environment of host tissues or extracellular matrix supporting regular physiology of different types of cells.^25^, ^26^, ^27^ The adjustable mechanical properties, logic controlled delivery behavior, and ultrahigh release precision render CCS an effective and mechanically smart scaffold for the applications in controlling cell fate,^28^ repairing tissue damage,^29^ and regenerating tissues.^30^

We developed a low‐temperature foaming technique that efficiently fabricates the sea sponge‐inspired hierarchical pore structures in ceramic‐based scaffold with feasibility to load molecules and cells in situ (Figure 1b and Figure S1, Supporting Information). In order to imitate the composite nature of sea sponge's skeleton and aim at tissue regeneration applications, bioactive HA and gelatinized cornstarch were selected as bioceramic and organic phases, respectively. The cornstarch also acts as a foaming agent for the stabilization of the porous structure. This foaming technique is applicable at relatively low temperatures ranged from ambient temperature to 90 °C, depending on the gelatinization temperatures of starch and the requirement of cargo (i.e., drug, protein, cell, etc.). The ceramic–starch foam can be readily cut or machined into any shape and form robust self‐standing CCS after air‐drying or freeze‐drying (Figure 1c). Dehydrated CCS generally has a porosity >85% (Figures S2 and S3, Supporting Information) and comprises of a three‐hierarchy pore structure with pore sizes varied from the millimeter to micrometer range (Figure 1d–f), which highly mimics the porous structure of the leuconoid sponge. The first hierarchy is comprised of spherical major pores with a narrow size distribution from ≈500 µm to ≈1 mm (Figure 1d), matching the dimension of the leucon's choanocyte chamber (Figure 1g). The sizes of major pores in this range are also desirable for bone ingrowth and regeneration.^31^, ^32^ The second hierarchy consists of interconnected pores with mainly open circular windows of 50–100 µm in diameter on the walls of the major pores (Figure 1e), similar to the sizes of incurrent canals and apopyles in leucon. The third hierarchy corresponds to a large amount of micrometer‐sized pores (<10 µm) uniformly residing on the walls of the major spherical pores (Figure 1f). Such minor pores resemble the interstices between choanocytes and, like the sponges using such interstices to trap food particles and bacterial cells, can serve as reservoirs for loading molecules and cells (Figure 1g).

Due to the structural and phase combination of hydrophilic HA particles and water‐absorbable starch, CCS exhibits moisture‐dependent mechanical properties that are attractive for on‐demand delivery and regenerative medicine. CCS with various moisture contents shows a viscoelastic stress–strain response (**Figure** **2**a). Most notably, air‐dried CCS with 83 wt% HA content and 87% porosity (moisture content <5%) is found to have remarkable compressive strength (σ_c_ = 1.20 ± 0.01 MPa) and modulus (*E*_c_ = 57.1 ± 1.3 MPa), which are much greater than the mechanical properties reported in porous systems of poly(d, Llactide‐co‐glycolide)/nano HA (*E*_c_ = 4.56 ± 0.3 MPa),^33^ poly(l‐lactic acid)/HA (*E*_c_ = 10.87 ± 3.20 MPa),^34^ and HA coated with polycaprolactone (σ_c_ = 0.57 ± 0.09 MPa).^35^ The compressive strength of CCS with low moisture content (e.g., <10%) is also comparable to strengths of demineralized and deproteinized trabecular bovine femur (DMB and DPB, Figure 2a),^36^ which are clinically used for bone substitution and regeneration.

**Figure 2:**
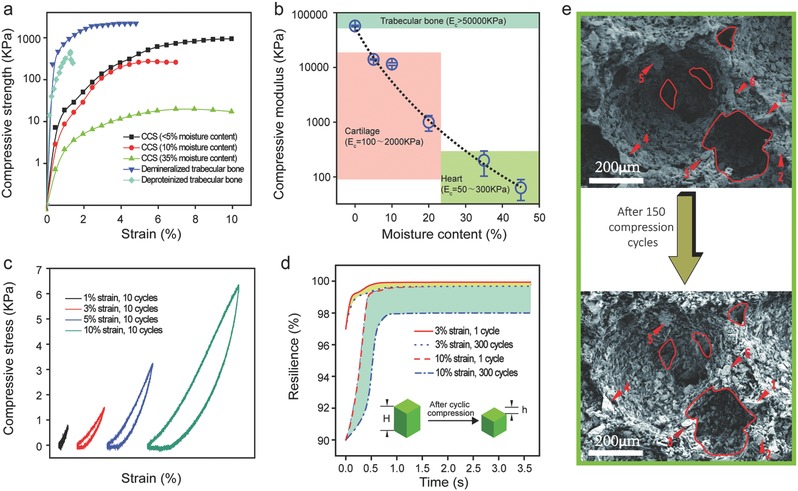
Moisture‐dependent mechanical properties of CCS. a) Compressive stress–strain relations of CCS (ceramic content = 83 wt%) with different moisture contents (<5, 10, and 35 wt%), comparing to that of demineralized trabecular bovine femur (DMB) and deproteinized trabecular bovine femur (DPB).^36^ b) Moisture‐dependent compressive modulus (*E*_c_) of CCS in comparison to that of different tissues. c) Compression–decompression loop of CCS with 45% moisture content subjected to different strains. d) Resilience versus recovery time relation of CCS with 45% moisture content subjected to different compressive cycles and strains (Resilience = [(*H* − *h*)/*H*] × 100%). e) SEM images showing the microstructure of CCS (ceramic content = 86 wt%) before and after 150 compression cycles (max. strain = 10%). The red lines outline pore morphology and the numbers indicate large ceramic particles, both of which remain unchanged after compression.

The high mechanical strengths of CCS are probably attributed to the reinforcing effect of starch molecules on ceramic particles. A close‐up examination of CCS pore walls reveals the structural interlocks between ceramic particles and starch molecules (Figure 1f). Gelatinized starch molecules form a 3D network grasping and binding HA particles that consist of ≈80 wt% of CCS, which is a mimicry of sea sponge's skeleton of mesohyl matrix embedded with mineral spicules and/or spongin fibers. The interlocking effects between ceramic particles and starch networks result in a strong cohesion between starch molecules and neighboring ceramic particles via a “bridging mechanism” (arrows in Figure 1f), ensuring the high robustness of CCS even at high ceramic content and porosity up to 86 wt% and 88%, respectively (Figure S3, Supporting Information). Interestingly, the same CCS can be sintered at 1250 °C for 2 h to obtain a porous ceramic scaffold with a porosity of 75% but its compressive strength and modulus drop to 0.18 and 11.6 MPa, respectively (Figure S3, Supporting Information). This decrease indicates that gelatinized starch in CCS actually reinforces the porous structure of HA rather than deteriorates its mechanical properties, agreeing to what has reported in the non‐porous HA/starch system.^37^

Static mechanical properties of CCS can be adjusted dramatically as the moisture in the sponge changes (Figure 2a,b) and its compressive modulus decreases exponentially to the range of kilopascal when the moisture increases to 35 wt%. This adjustability enables CCS to match the compliance of target tissues, which has been a persistent drawback of ceramic‐based 3D scaffolds in the applications of controlled release and tissue regeneration.^38^ CCS can match the compliances of trabecular bone (*E*_c_ > 50 MPa), cartilage (*E*_c_ = 0.1–2 MPa), and heart tissue (*E*_c_ = 0.01–0.02 MPa) at moisture contents of 3, 15–35, and 45 wt%, respectively (Figure 2b). Given that internal moisture (water) contents in trabecular bone, cartilage, and heart muscle are ≈10, 60–80, and 70 wt%, respectively,^17^ CCS implanted to these target tissues are expected to equalize its moisture content with surrounding tissues and eventually render its stiffness compatible with that of the host tissue.

More importantly, like a real sea sponge, CCS with elevated moisture contents (usually >10 wt%) becomes remarkably resilient at different amounts of deformation. Figure 2c demonstrates the recoverable loading–unloading loops of CCS measured at the 11th compression cycle of different strains (i.e., CCS experienced ten compression cycles before the measurement), revealing a reversible elasticity at 1% strain while maintaining a full recover ability up to 10% strain. The compliance of CCS, however, is preserved at different compressive strains (≈60 kPa in Figure 2c) and is much greater than the well‐known hydrogels^39^ or ferrogel^40^ reported for drug or cell delivery. Resilience of CCS remains at 99.5% and 98% after 300 cycles of compression at 3% and 10% strains, respectively (Figure 2d). The remarkable resilience of CCS is also reflected by its ultrafast recovery when the compression is removed (Figure 2d and Movie S1, Supporting Information), taking only a few hundred milliseconds to recover even after 300 compression cycles of 10% strain. Direct observation before and after 150 compression cycles suggests CCS preserves its porous structure, with positions of pore ceramic particles remaining identical (Figure 2e), demonstrating a high robustness and stability for mechanically modulated applications.

The great resilience and adjustable compliance of CCS are predominantly rooted in high viscoelasticity and pliability of the gelatinized starch chain network which can hydrate or dehydrate reversibly. Abundant hydroxyl groups in both HA and starch can form a large quantity of hydrogen bonds in the presence of water, which plasticizes the starch molecules to achieve a substantial interfacial stability between ceramic and starch network, restricting the movement and rotation of ceramic particles and eventually maintaining the integrity of CCS during cyclic deformation.

Similar to sea sponge's delicate transportation of multi‐scale food, gas, and waste particles in its hierarchical body system, CCS also allows accurate releases of various cargos from molecules to proteins to cells by mechanical modulation. Mechanically modulated cargo release by CCS exhibits a binary‐stage pattern in deionized water (DI water) (equivalent to ≈66 wt% moisture content). Model molecules of bromophenol blue (BPB) release only 0.27–0.35 µg cm^−3^ from CCS at compressive strains <2% for 20 consecutive compression cycles, but the release rapidly quintuples to and saturates at 1.47–1.58 µg cm^−3^ when the strain rises above 3% (**Figure** **3**a and Movie S2, Supporting Information). This binary‐stage release resembles a mechanically modulated digital gate whose low and high release states correspond to 0 and 1, respectively, with a threshold at 3% compressive strain.

**Figure 3:**
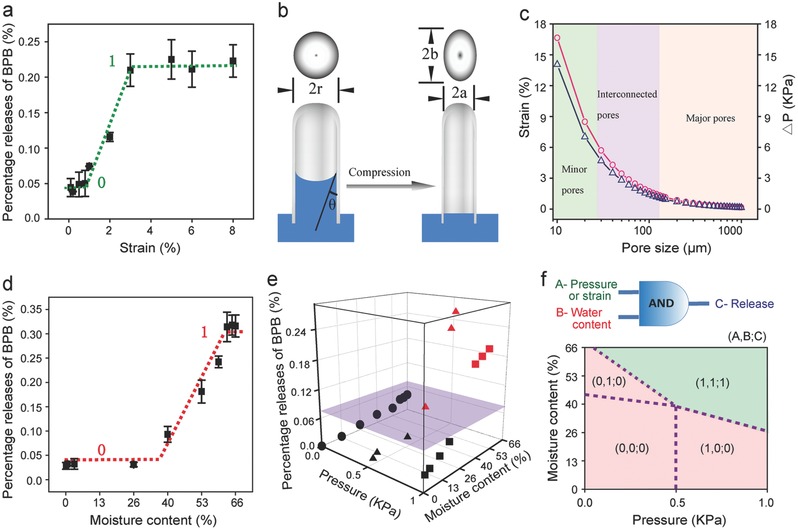
Mechano‐active CCS for AND logic release. a) Cumulative release of BPB from CCS at different strains. CCS has a moisture content of 66% and is subjected to ten compression cycles at each strain. b) Simplified cylindrical pore model for the analysis of water expelled from pores in CCS. Before compression, the pore has a circular cross section with a diameter of 2*r* and the hydrophilic liquid has a contact angle of θ. After compression, the deformed cylindrical pore has an elliptical cross section with two axes of (a) and (b). c) Estimated counter pressure (∆*P*, blue triangles) and compressive strains (ε_repel_, red circles) as a function of pore size. d) Cumulative release of BPB from CCS at different moisture contents. CCS is subjected to ten compression cycles at a pressure of 0.5 kPa. e) 3D diagram of AND logic release in CCS, where the vertical axis is the cumulative release of BPB and the horizontal axes are moisture content and pressure, respectively. CCS is subjected to ten compression cycles at each point. f) Logic map showing True (1) and False (0) relations between dual inputs of A) pressure/strain and B) moisture content, and C) output of release. Red zone represents C = 0 (False) and green zone represents C = 1 (True). Dot lines represent the thresholds of logic gates A and B.

The hierarchical pore structure of CCS allows liquids to flow into the interior of the sponge and reach the large surface areas of pore walls where cargos (e.g., drugs or cells) are preserved. The high resilience of CCS, on the other hand, allows the formation of strong water convection and shear force in the structure when compressed,^2^ assisting the release of cargos. Theoretically, the cargo release capability enabled by fluid convection should be a function of the counter pressure applied to squeeze the fluid out of the pores against capillary pressure. Implied by the Young‐Laplace equation for a simplified, cylindrical pore structure (Figure 3b), this counter pressure (Δ*P*) is inversely proportional to the pore size for a specific liquid and was estimated to be on the order of kilopascal for water in the pores with sizes from 10–1000 µm (Figure 3c; see the Supporting Information for calculation). The counter pressure presumably originates from the shear of pores under external compression and the strain required for completely repelling the water (ε_repel_) can be estimated (Figure 3c; see the Supporting Information for calculation). Assuming average sizes of 50–100 µm for the interconnect pores in CCS (also canals and apopyles in sea sponge), the minimum of ε_repel_ is calculated to be 2.9%, which agrees with the threshold of 3% in the strain gate observed in experiment (Figure 3a). In addition, theoretical predictions of ε_repel_ (0.2%–17%) in a range of pore sizes are close to the values used in the experiments (3%–20%) to achieve satisfactory delivery of drugs or cells.

Besides the pressure/strain gate, CCS has another binary gate of moisture which restricts the release down to <0.25 µg cm^−3^ of CCS (20 cycles cumulative) at water contents <40 wt% while allowing up to a ten‐fold increase at water contents >45 wt% (Figure 3d and Movie S3, Supporting Information). The binary gates of strain (or pressure) and moisture together construct an AND logic gate, by which the cargo is only released when true inputs from both binary gates are met (Figure 3e). The logic map of dual‐controlled release is shown in Figure 3f, revealing all AND logic conditions for CCS to deliver or retain cargos, which, interestingly, also reveals an interrelated true‐false boundary between strain (or pressure) and moisture gates. For example, the true input of the pressure gate required less than half of the pressure (1 kPa vs. 0.5 kPa) when the moisture threshold increased from 26 to ≈60 wt%. Based on this logic map, cargos released by CCS can be accurately controlled by selecting appropriate strain (pressure) and water content. Therefore, at stable water contents, the amount and intermittence of release can be readily tailored by the strain/pressure, realizing high‐precision temporal, spatial and even programmable controls on drug or cell delivery.

Ultrahigh precision logic delivery of molecules and cells are achieved by CCS under mechanical stimuli. CCS loaded with water‐insoluble BPB (payload 700 µg cm^−3^) and subjected to compression (state 1) and decompression (state 0) cycles (“on/off” status) of 3% strain exhibits linear cumulative release proportional to the cycle numbers (**Figure** **4**a). More importantly, the release amount modulated by 3% compressive strain is highly repeatable in every cycle and reaches a remarkable precision of ≈70 ng per cycle cm^−3^ of CCS (Figure 4b). Meanwhile, background release without mechanical stimuli (“off” status) is less than 10 ng per cycle cm^−3^. Besides hydrophobic molecules, ultrahigh precision is maintained in water‐soluble hydrophilic molecules of bovine serum albumin (BSA), reaching a precision of 150–200 ng per cycle per cm^3^ CCS (Table S1, Supporting Information). To our best knowledge, this on‐demand, nanogram precision delivery of molecules by porous systems is reported for the first time and the release precision is much higher compared to other macroporous system reported recently (Table S2, Supporting Information).^41^, ^42^, ^43^ This mechanically induced ultrahigh release precision of ≈10 ng mL^−1^ for proteins is more superior than an electrically controlled release by a hydrogel system reported recently, which releases lysozyme protein at a precision of 2000 ng mL^−1^ per cycle for a hydrogel layer much less than 1 cm^3^ (≈1.4 mm^3^).^44^

**Figure 4:**
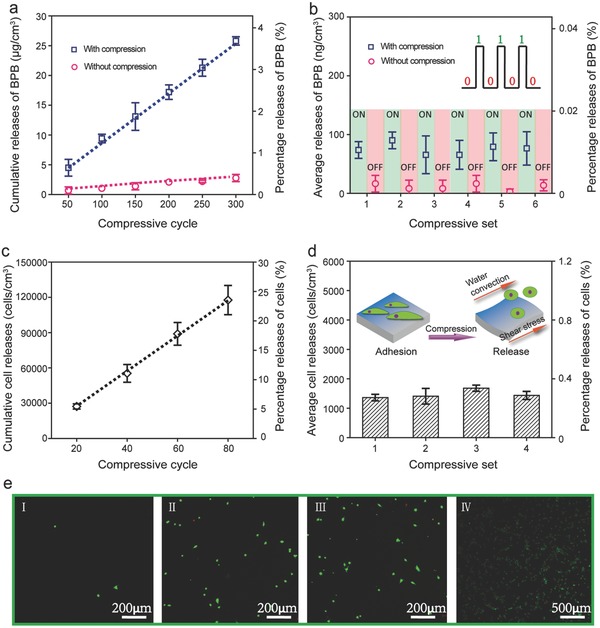
Mechano‐active CCS for cyclic delivery of molecules and cells. a) Cumulative release of BPB from CCS subjected to 300 consecutive compression cycles (blue boxes) and the release from CCS without compression (red circles, tested at the same time periods). The second *y*‐axis denotes the percentage of released BPB compared to the total amount loaded in CCS. b) The average amount of BPB per cycle released from a unit volume of CCS during compression (“ON”, blue boxes) and the release from CCS without compression (“OFF”, red circles). CCS is tested for six consecutive sets and each set contains 50 compression cycles. Compressive strain and moisture content are 3% and 66%, respectively, for all the CCS tested. c) Cumulative number of cells released from CCS subjected to 80 consecutive compression cycles. The second *y*‐axis denotes the percentage of released cells compared to the total amount loaded in CCS. d) The average number of cells per cycle released from a unit volume of CCS. CCS is tested for four consecutive sets and each set contains 20 compression cycles. Compressive strain and moisture content are 20% and 66%, respectively, for all the CCS tested. e) Fluorescence images of cells released from CCS I) without compression, II) after the first and III) the fourth compression sets, and IV) viable cells remaining in the CCS after 80 consecutive compression cycles. In the fluorescence images, live cells are stained green and dead cells are stained red.

CCS, infiltrated with fibroblasts (payload 5 × 10^5^ cells cm^−3^) and allowed cell adhesion for 4 h, demonstrates a similar linear cumulative release pattern to the BPB release and a repeatable on‐off release for the compression cycles of 20% strain (Figure 4c,d). The precision of cell release per cycle reaches 1400 cells per cm^3^ CCS, tested in 10 mL of culture medium (Figure 4d), while its background release without compression is almost negligible (Figure 4e). In addition, the released fibroblasts retain high viability after tens of cycles of 20% strain, even after the CCS is completely smashed (Figure 4e). It is worth mentioning that the mechanically modulated, ultrahigh release precision of 1400 cells mL^−1^ per cycle by 1 cm^3^ CCS outperforms that of any on‐demand delivery systems so far.^45^, ^46^

In contrast to conventional strategy,^47^, ^48^ we did not use additional linkers or adhesive proteins to tether the hydrophobic BPB, hydrophilic albumin, or cells. Retention of the cargos in CCS probably relies on weak bonds like hydrogen bonds and intermolecular forces since both HA and starch molecules have an abundant amount of surface hydroxyl groups. The above results indicate that the weakly bonded cargos can be readily released when the gates of moisture and strain are open and vice versa. For loading highly water‐soluble entities or special cargos in CCS, additional linkage is certainly useful and can be designed depending on specific applications.

In summary, on‐demand ultrahigh precision logic delivery is achieved in mechanically modulated CCS that has a three‐level hierarchy of porosity and resilience inspired by leuconoid sponge. The capacity of logic delivery of molecules and cells with ultrahigh precision and repeatability makes CCS a truly on‐demand delivery system that enables temporal, spatial, and quantitative controls of cargos via mechanical stimuli. CCS is also a versatile platform that can mechanically adapt to the compliance of host tissue or environment by adjusting its moisture content. Such bioinspired mechano‐active ceramic sponges are thus promising for delicate programmable and gate controlled delivery, opening a wide range of applications from environmental to biomedical applications.^49^, ^50^, ^51^, ^52^, ^53^, ^54^ For instance, CCS is expected to be an ideal tissue engineering scaffold for bones, cartilage, and muscle repair, where patient's movement acts as external stimuli to actively regulate the highly precise release kinetics of drugs (e.g., bone morphological proteins, antibiotics, anti‐osteoporosis drugs, etc.) or stem cells. CCS may also rely on intrinsic mechanical effects, such as hemokinesis, heartbeat, and peristalsis to achieve precise temporal control of drugs or cell releases. Additional applications of CCS also include logic sensors and programmable delivery devices for electrical, energy, and environmental engineering.

## Experimental Section

*Materials*: Food‐grade cornstarch was purchased from Weimeisi Co., Ltd (Shanghai, China). HA was synthesized in house according to a method described elsewhere.^55^ Triton X‐100 was purchased from Sinopharm Chemical Reagent Co., Ltd. (Shanghai, China). BPB was procured from Sigma (St. Louis, MO).

*Fabrication of CCS*: Cornstarch and DI water was homogeneously mixed in a beaker to obtain 10 wt% starch suspension. Different amounts of HA powders were then added into the starch suspension and mixed uniformly to form ceramic slurries. For different purposes, the solid contents of HA (the ratio of HA mass to total solid mass) varied from 71.4% to 85.9%. The slurry was later heated to 90 °C in a water bath and a surfactant of Triton X‐100 was added. The heated suspension became a highly viscous gel and then was vigorously stirred by an overhead stirrer till air bubbles fully infiltrated into the suspension to obtain foam. The foam was removed from the water bath, cooled down to room temperature, and then set for 24 h. The foam was further dried to porous composite scaffolds with desired moisture contents, depending on different applications. Porous HA ceramics were sintered from dried CCS with 80% ceramic content. CCSs with the dimension of 15 × 15 × 15 mm^3^ were sintered at 1250 °C using a muffle furnace (SJKQ‐1700, Dingan Tec, Suzhou) for 2 h.

*Preparation of BPB‐ and BSA‐Loaded CCS*: 8 g starch, different amounts of hydroxyapatite and appropriate volume of surfactant were mixed with 72 mL 0.1 wt% BPB solution in a 200 mL beaker to obtain the uniform slurry, and then the slurry was heated to 90 °C in a water bath to allow starch to gelatinize. After that the slurry was foamed and set for 24 h to get the stable porous network. For BSA loading, the BPB solution was replaced by DI water. After foaming at 90 °C, the beaker was placed at room temperature until the temperature of the slurry dropped to 50 °C, 3.5 g of BSA (Sigma, St. Louis, MO) was added in the beaker and the slurry was foamed. The foam was set for 24 h to get the stable porous network. BPB‐ and BSA‐loaded CCS could be cut to different shapes and dried by dehydration (dried by gradient of ethanol solution) or freeze drying (−54 °C) depending on different applications.

*Characterization of CCS*: Microstructure and morphology of CCS were characterized by scanning electron microscopy (SEM, FEI Quanta 250, acceleration voltage of 1.5 kV under a vacuum of 1.56 × 10^−4^ Pa). The sample was fractured and the facture surface for observation was sputtering‐coated with Au‐Pd. Dried CCSs with the dimension of 10 × 10 × 10 mm were scanned at the speed of 0.7° s^−1^ by Micro‐Computed Tomography (Micro‐CT, Skyscan1176) with the precision of 8.8 µm, and then a 3D microstructure of CCSs was obtained by reconstructions of micro‐CT data. Apparent density (ρ_app_) of CCS and porous HA ceramic was calculated by its weight and volume that can be directly measured. Theoretical density (ρ_th_) of the porous composites was calculated by the densities of the HA and starch according to their proportions in the composites. The porosity (*p*) of CCS was thus calculated by the equation

(1) $$p=\left(1-\frac{\rho_{\mathrm{app}}}{\rho_{\mathrm{th}}}\right)\;\;\;\times \;\;100\%$$

*Mechanical Characterization*: For uniaxial compression tests, CCS were cut into cubes with dimension of 10 × 10 × 10 mm^3^ and tested on a mechanical tester (HY‐1080, testing range 0–500 N with precision of 0.01 N, Hengyi company, Shanghai) operating at a crosshead speed of 1 mm min^−1^. From the stress–strain curve, the maximum stress before failure was determined as compressive strength and the linear range in the stress curve before failure was used to calculate compressive modulus. For the testing of the compressive loops of CCS, CCSs with 45% moisture content were cut into cubes with dimension of 10 × 10 × 10 mm^3^ and the cubes were pressed to a desired strain of CCS (1%, 3%, 5%, and 10%) and then unloaded to their initial position. Resilience was characterized using a CCS sample with 45% water contents using a microscope. A CCS cube was pressed to designated strains (3% and 10%), and released, and this process was video‐recorded by a microscope. The resilience was then calculated by comparing the position of the rebounding surface to its initial position.

*BPB Release Behavior of CCS*: Release behavior of CCS loaded with BPB was studied by compressing CCS cubes (10 × 10 × 10 mm^3^) at different strains (0.1%, 0.2%, 0.5%, 0.8%, 1%, 2%, 3%, 5%, 6%, and 8%) in a series of ethanol/water solution (water contents 0, 20%, 40%, 60%, 80%, and 100%). After pressing for twenty times, the solution containing released BPB was retrieved and spectrophotometrically measured on a microplate reader (at 450 nm on BioTek MQX200R). Optical densities (OD) of the solution were compared to a standard OD curve of solution containing known concentrations of BPB (see Figure S4 and Supporting Information for more information). Meanwhile, a CCS cube that was placed in the same ethanol/water solution but not pressed was used as a control to calculate the BPB released from CCS without mechanical stimulation. For cyclic release tests, CCS was compressed at designated strains for 50 consecutive cycles (defined as one set of compression) and the solution containing released BPB was removed for spectrophotometical measurement. Then test resumed for another set and repeated up to six sets in total. A parallel test of the CCS without compression was used to determine the background release. The amount of BPB released in each set (background release was subtracted) was averaged by 50 cycles and then the total volume of CCS to obtain the net release amount per cycle per cm^3^ of CCS. All the releases tests were repeated at least three times.

*Calculation of the Amount of BPB Released from CCS*: 100 µL of BPB solution with different concentration was placed in a 96‐well plate, and then the solution was spectrophotometrically measured on a microplate reader at a wavelength of 450 nm (BioTek MQX200R). The as‐measured OD value was corrected by subtracting the background of DI water. After that, the standard curve of BPB concentrations versus OD values was obtained. The experiments were repeated at least three times. In order to calculate the amounts of the BPB released from the porous composite scaffolds with different compressive strains, the as‐measured OD value was corrected by subtracting the background of DI water. The concentration of the BPB solution (c) could be calculated by comparing it with the standard curve. Assuming the total volume of the BPB solution released and the volume of the CCS cubes were *v*_1_ and *v*_2_, respectively. Then the amount of BPB released from per cm^3^ CCS could be calculated as

(2) $$m\;\;=\;\;\frac{cv_{1}}{v_{2}}$$

*Measurement of the Amount of BSA Released from CCS*: For cyclic release of BSA test, BSA‐loaded CCS was compressed at designated strains (3%) for ten consecutive cycles (defined as one set of compression) and the solution containing released BAS was then removed for spectrophotometrical measurements (100 µL to 96‐well plate, 570 nm) before treated with a Micro BCATM Protein Assay Kit (Prod#23235, Thermo Scientific). After, the test was resumed for another set and repeated up to 15 sets in total. A parallel test of the CCS without compression was used to determine the background release. The amount of BSA was measured following the instructions of the protein kit, and then the amount of BSA released in each set subtracted the background release was averaged by ten cycles and then total volume of CCS to obtain the net release amount per cycle per cm^3^ of CCS. All the releases tests were repeated at least three times.

*Cell Release Test*: Cell‐loaded CCS was immersed in a cell culture medium (Dulbecco's modified eagle medium (DMEM) with 10% fetal bovine serum (FBS) and 1% penicilin‐streptomycin (P/S)) and then subjected to cyclic compressions at a strain of 20%. CCS was compressed for 20 consecutive cycles (defined as one set of compression) and the cell culture medium containing released cells was removed for a 24 h culture and then cell count by the Live/Dead Viability/Cytotoxicity Kit (Thermo Fisher Scientific, L‐3224) according to its instructions. Then test resumed for another set and repeated up to four sets in total. A parallel test of the CCS without compression was used to determine the background release of cells. Live/Dead cells after staining were imaged by fluorescence microscopy (ZEISS, AxioCamHRc) and the remaining number of live cells was counted. The number of live cells released in each set subtracted by the number of background release, averaged over 20 cycles and then total volume of CCS to obtain the net released cell number per cycle per cm^3^ of CCS. All the releases tests were repeated three times.

## Supporting information

As a service to our authors and readers, this journal provides supporting information supplied by the authors. Such materials are peer reviewed and may be re‐organized for online delivery, but are not copy‐edited or typeset. Technical support issues arising from supporting information (other than missing files) should be addressed to the authors.

Supplementary

Supplementary

Supplementary

Supplementary

## References

[1] C. A. Cezar , S. M. Kennedy , M. Mehta , J. C. Weaver , L. Gu , H. Vandenburgh , D. J. Mooney , Adv. Healthcare Mater. 2014, 3, 1869. 10.1002/adhm.201400095 PMC4227916 24862232

[2] X. Zhao , J. Kim , C. A. Cezar , N. Huebsch , K. Lee , K. H. Bouhadir , D. J. Mooney , Proc. Natl. Acad. Sci. USA 2011, 108, 67. 21149682 10.1073/pnas.1007862108 PMC3017202

[3] N. Huebsch , C. J. Kearney , X. Zhao , J. Kim , C. A. Cezar , Z. Suo , D. J. Mooney . Proc. Natl. Acad. Sci. USA 2014, 111, 9762. 24961369 10.1073/pnas.1405469111 PMC4103344

[4] J. Chen , H. Chen , P. Li , H. Diao , S. Zhu , L. Dong , R. Wang , T. Guo , J. Zhao , J. Zhang , Biomaterials 2011, 32, 4793. 21489619 10.1016/j.biomaterials.2011.03.041

[5] W. Ji , Y. Sun , F. Yang , J. J. J. P. van den Beucken , M. W. Fan , Z. Chen , J. A. Jansen , Pharm. Res. 2011, 28, 1259. 21088985 10.1007/s11095-010-0320-6 PMC3098998

[6] P. B. Malafaya , G. A. Silva , R. L. Reis , Adv. Drug Delivery Rev. 2007, 59, 207. 10.1016/j.addr.2007.03.012 17482309

[7] J. S. Park , D. G. Woo , B. K. Sun , H. Chung , S. J. Im , Y. M. Choi , K. Park , K. M. Huh , K. Park , J. Controlled Release 2007, 124, 51. 10.1016/j.jconrel.2007.08.030 17904679

[8] X. Hu , S. Liu , G. Zhou , Y. Huang , Z. Xie , X. Jing , J. Controlled Release 2014, 185, 12. 10.1016/j.jconrel.2014.04.018 24768792

[9] J. Siepmann , F. Siepmann , J. Controlled Release 2012, 161, 351. 10.1016/j.jconrel.2011.10.006 22019555

[10] A. Godierfurnemont , T. P. Martens , M. S. Koeckert , L. Q. Wan , J. Parks , K. Arai , G. Zhang , B. I. Hudson , S. Homma , G. Vunjaknovakovic , Proc. Natl. Acad. Sci. USA 2011, 108, 7974. 21508321 10.1073/pnas.1104619108 PMC3093484

[11] L. M. Ensign , R. A. Cone , J. Hanes , Adv. Drug Delivery Rev. 2012, 64, 557. 10.1016/j.addr.2011.12.009 PMC3322271 22212900

[12] Y. Qiu , K. Park , Adv. Drug Delivery Rev. 2001, 64, 49. 10.1016/s0169-409x(01)00203-4 11744175

[13] J. Kost , R. Langer , Adv. Drug Delivery Rev. 2001, 64, 327. 10.1016/s0169-409x(00)00136-8 11259837

[14] R. de la Rica , D. Aili , M. M. Stevens , Adv. Drug Delivery Rev. 2012, 64, 967. 10.1016/j.addr.2012.01.002 22266127

[15] R. Kurapati , A. M. Raichur , Chem. Commun. 2012, 49, 734. 10.1039/c2cc38417e 23232330

[16] M. N. Holme , I. A. Fedotenko , D. Abegg , J. Althaus , L. Babel , F. Favarger , R. Reiter , R. Tanasescu , P. Zaffalon , A. Ziegler , B. Muller , T. Saxer , A. Zumbuehl , Nat. Nanotechnol. 2012, 7, 536. 22683843 10.1038/nnano.2012.84

[17] C. R. Ethier , C. A. Simmons , Introductory Biomechanics: From Cells to Organisms, Cambridge University Press, NY 2007.

[18] K. Lee , M. C. Peters , K. W. Anderson , D. J. Mooney , Nature 2000, 408, 998. 11140690 10.1038/35050141

[19] D. C. Hyun , G. D. Moon , C. J. Park , B. S. Kim , Y. Xia , U. Jeong , Angew. Chem., Int. Ed. 2011, 50, 724. 10.1002/anie.201004838 21226163

[20] J. F. Doerner , S. Febvay , D. E. Clapham , Nat. Commun. 2012, 3, 990. 22871809 10.1038/ncomms1999 PMC3651673

[21] S. P. Leys , A. Hill , Adv. Mar. Biol. 2012, 62, 1. 22664120 10.1016/B978-0-12-394283-8.00001-1

[22] E. E. Ruppert , R. S. Fox , R. D. Barnes , Invertebrate Zoology: A Functional Evolutionary Approach, Brooks/Cole‐Thompson, Belmont, USA 2004.

[23] S. P. Leys , D. I. Eerkesmedrano , Biol. Bull. 2006, 211, 157. 17062875 10.2307/4134590

[24] R. W. Thacker , M. C. Diaz , A. Kerner , R. Vigneslebbe , E. Segerdell , M. Haendel , C. J. Mungall , J. Biomed. Semant. 2014, 5, 1. 10.1186/2041-1480-5-39 PMC4177528 25276334

[25] B. Hu , W. Shi , Y. Wu , W. Leow , P. Cai , S. Li , X. Chen , Adv. Mater. 2014, 26, 5786. 25066463 10.1002/adma.201402489

[26] P. Cai , M. Layani , W. Leow , S. Amini , Z. Liu , D. Qi , B. Hui , Y. Wu , A. Miserez , S. Magdassi , X. Chen , Adv. Mater. 2016, 28, 3102. 26913959 10.1002/adma.201505300

[27] C. Tay , Y. Wu , P. Cai , N. Tan , S. S. Venkatraman , X. Chen , L. Tan , NPG Asia Mater. 2015, 7, e199.

[28] O. Chaudhuri , L. Gu , D. D. Klumpers , M. C. Darnell , S. A. Bencherif , J. C. Weaver , N. Huebsch , H. Lee , E. Lippens , G. N. Duda , D. J. Mooney , Nat. Mater. 2015, 15, 326. 26618884 10.1038/nmat4489 PMC4767627

[29] F. Yu , X. Cao , Y. Li , L. Zeng , J. Zhu , G. Wang , X. Chen , Polym. Chem. 2014, 5, 5116.

[30] N. Huebsch , E. Lippens , K. Lee , M. Mehta , S. T. Koshy , M. C. Darnell , R. M. Desai , C. M. Madl , M. Xu , X. Zhao , O. Chaudhuri , C. Verbeke , W. S. Kim , K. Alim , A. Mammoto , D. E. Ingber , G. N. Duda , D. J. Mooney , Nat. Mater. 2015, 14, 1269. 26366848 10.1038/nmat4407 PMC4654683

[31] V. Karageorgiou , D. L. Kaplan , Biomaterials 2005, 26, 5474. 15860204 10.1016/j.biomaterials.2005.02.002

[32] L. Sicchieri , G. E. Crippa , P. T. De Oliveira , M. M. Beloti , A. L. Rosa , J. Tissue Eng. Regener. Med. 2012, 6, 155. 10.1002/term.422 21446054

[33] S. Kim , M. S. Park , O. Jeon , C. Y. Choi , B. Kim , Biomaterials 2006, 27, 1399. 16169074 10.1016/j.biomaterials.2005.08.016

[34] P. X. Ma , R. Zhang , G. Xiao , R. T. Franceschi , J. Biomed. Mater. Res. 2001, 54, 284. 11093189 10.1002/1097-4636(200102)54:2<284::aid-jbm16>3.0.co;2-w

[35] J. Zhao , L. Guo , X. Yang , J. Weng , Appl. Surf. Sci. 2008, 255, 2942.

[36] P. Chen , J. Mckittrick , J. Mech. Behav. Biomed. Mater. 2011, 4, 961. 21783106 10.1016/j.jmbbm.2011.02.006

[37] H. Liu , Y. Guan , D. Wei , C. Gao , H. Yang , L. Yang , J. Biomed. Mater. Res. Part B 2015, 104, 615. 10.1002/jbm.b.33434 25953516

[38] A. R. Amini , C. T. Laurencin , S. P. Nukavarapu , Crit. Rev. Biomed. Eng. 2012, 40, 363. 23339648 10.1615/critrevbiomedeng.v40.i5.10 PMC3766369

[39] A. D. Augst , H. J. Kong , D. J. Mooney , Macromol. Biosci. 2006, 6, 623. 16881042 10.1002/mabi.200600069

[40] V. Zamoramora , P. Soares , C. Echeverria , R. Hernandez , C. Mijangos , Gels 2015, 1, 69. 10.3390/gels1010069 PMC6318601 30674166

[41] A. C. Richards Grayson , I. S. Choi , B. M. Tyler , P. P. Wang , H. Brem , M. J. Cima , R. Langer , Nat. Mater. 2003, 2, 767. 14619935 10.1038/nmat998

[42] N. S. Satarkar , J. Z. Hilt , J. Controlled Release 2008, 130, 246. 10.1016/j.jconrel.2008.06.008 18606201

[43] P. Roy , A. Shahiwala , J. Controlled Release 2009, 134, 74. 10.1016/j.jconrel.2008.11.011 19105973

[44] R. Feiner , L. Engel , S. Fleischer , M. Malki , I. Gal , A. Shapira , Y. Shacham‐Diamand , T. Dvir , Nat. Mater. 2016, 15, 679. 26974408 10.1038/nmat4590 PMC4900449

[45] M. Wirkner , J. M. Alonso , V. Maus , M. Salierno , T. T. Lee , A. J. Garcia , A. D. Campo , Adv. Mater. 2011, 23, 3907. 21618293 10.1002/adma.201100925

[46] C. L. Hastings , E. T. Roche , E. Ruiz‐Hernandez , K. Schenke‐Layland , C. J. Walsh , G. P. Duffy , Adv. Drug Delivery Rev. 2015, 84, 85. 10.1016/j.addr.2014.08.006 25172834

[47] S. Mura , J. P. Nicolas , P. Couvreur , Nat. Mater. 2013, 12, 991. 24150417 10.1038/nmat3776

[48] K. L. Kim , D. K. Han , K. Park , S. Song , J. Kim , J. Kim , H. Y. Ki , S. W. Yie , C. Roh , E. Jeon , D. Kim , W. Suh , Biomaterials 2009, 30, 3742. 19394079 10.1016/j.biomaterials.2009.03.053

[49] Y. K. Lai , L. X. Lin , F. Pan , J. Y. Huang , R. Song , Y. X. Huang , C. J. Lin , H. Fuchs , L. F. Chi , Small 2013, 9, 2945. 23420792 10.1002/smll.201300187

[50] Y. K. Lai , J. Y. Huang , Z. Q. Cui , M. Z. Ge , K. Q. Zhang , Z. Chen , L. F. Chi , Small 2016, 12, 2203. 26695122 10.1002/smll.201501837

[51] S. T. Wang , K. S. Liu , X. Yao , L. Jiang , Chem. Rev. 2015, 115, 8230. 26244444 10.1021/cr400083y

[52] K. S. Liu , M. Y. Cao , A. Fujishima , L. Jiang , Chem. Rev. 2014, 114, 10044. 24956456 10.1021/cr4006796

[53] H. Wang , B. W. Zhu , X. H. Ma , Y. Hao , X. D. Chen , Small 2016, 12, 2715. 27028213 10.1002/smll.201502906

[54] B. W. Zhu , H. Wang , W. R. Leow , Y. R. Cai , X. J. Loh , M. Y. Han , X. D. Chen , Adv. Mater. 2016, 28, 4250. 26684370 10.1002/adma.201504276

[55] A. Ebrahimpour , M. Johnsson , C. F. Richardson , G. H. Nancolla , J. Colloid Interface Sci. 1993, 159, 158.

