# Supplementary material for: Bioinspired Mechano‐Sensitive Macroporous Ceramic Sponge for Logical Drug and Cell Delivery
Source: Adv Sci (Weinh). 2017 Apr 27;4(6):1600410. doi: 10.1002/advs.201600410 (PMC5473326; doi:10.1002/advs.201600410)
Supplement: Supplementary file 1 — Supplementary [file ADVS-4-na-s001.pdf]

Copyright WILEY-VCH Verlag GmbH & Co. KGaA, 69469 Weinheim, Germany, 2017.

ADVANCED SCIENCE Open Access

Supporting Information

for *Adv. Sci.,* DOI: 10.1002/advs.201600410

Bioinspired Mechano-Sensitive Macroporous Ceramic Sponge for Logical Drug and Cell Delivery

Changlu Xu, Zhihao Wei, Huajian Gao, Yanjie Bai, Huiling Liu, Huilin Yang, Yuekun Lai, and Lei Yang**

Supporting Information

Bio-inspired Mechano-sensitive Macroporous Ceramic Sponge for Logical Drug and Cell Delivery

Changlu Xu, Zhihao Wei, Huajian Gao, Yanjie Bai, Huiling Liu, Huilin Yang, Yuekun Lai, and Lei Yang**

C. Xu, Z. Wei, H. Liu, Prof. H. Yang, Prof. L. Yang

Orthopaedic Institute, Department of Orthopaedics, the First Affiliated Hospital, Soochow University, Suzhou, Jiangsu 215006, P.R. China

E-mail: leiy@suda.edu.cn

Prof. H. Gao

School of Engineering, Brown University, Providence, RI 02912, USA

Prof. Y. Bai

School of Public Health, Medical College, Soochow University, Suzhou, Jiangsu 215123, P.R. China

Prof. Y. Lai

National Engineering Laboratory for Modern Silk, College of Textile and Clothing Engineering, Soochow University, Suzhou 215123, P.R. China

E-mail: yklai@suda.edu.cn

Prof. H. Gao, Prof. H. Yang, Prof. Y. Lai, Prof. L. Yang

International Research Center for Translational Orthopaedics (IRCTO), Soochow University, Suzhou, Jiangsu 215006, P.R. China

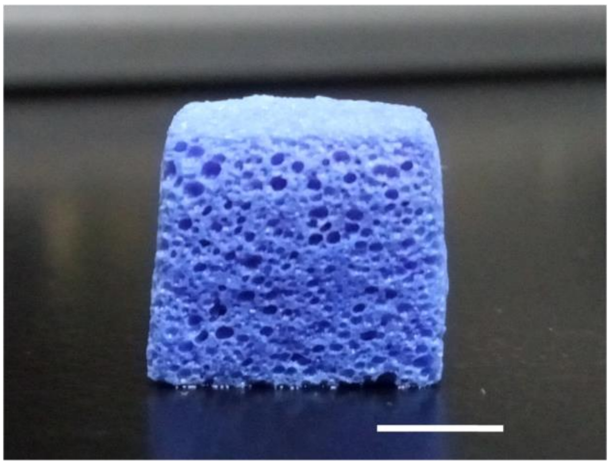

Figure S1. BPB molecules were loaded in the porous CCS in situ due to the relative moderate condition of the preparation of CCS. The simple material component, moderate pH and the relative low temperature could not damage the structure of the model drug. The in situ loading of the drug reduces the fabrication steps and improve the efficiency of the drug. Scale bar: 5 mm.

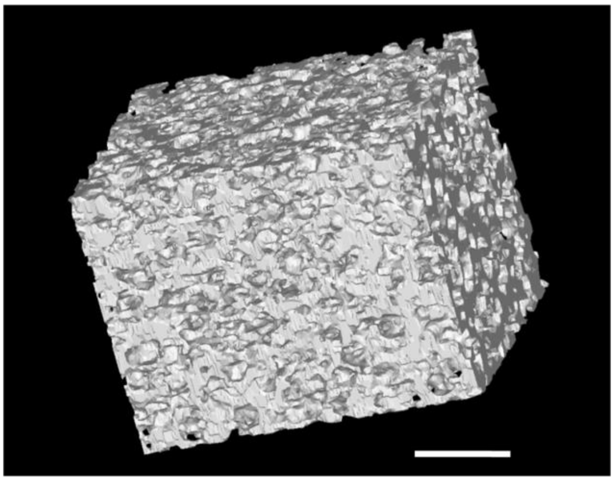

Figure S2. Three-dimensional microstructures obtained by reconstructions of micro-CT images suggested that CCS have high porosity and macro pores. Besides small molecules, these large pores were suitable for the loading and delivery of biological agents with large size like protein and cells. These large pores of CCS were determined by the foaming fabrication method, and the pore size depends on the property of the starch/ceramic suspension, including viscosity, temperature, density, surface tension, etc. Scale bar: 2 mm.

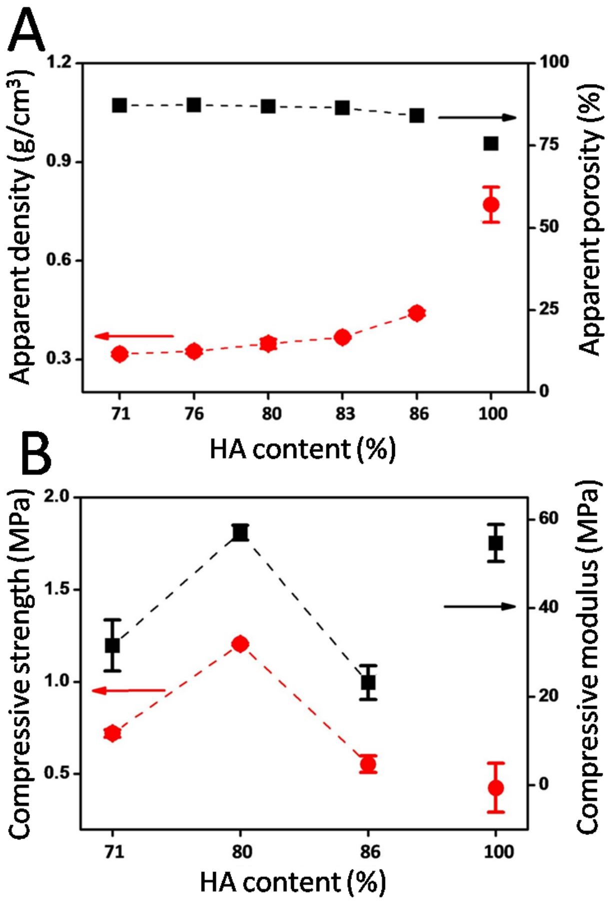

Figure S3. CCSs fabricated with foaming approach and porous HA ceramics have low apparent density and high porosity (A). Additionally, CCSs with high porosity have good mechanical properties, and were better than HA ceramics with lower porosity (B). Values represent mean and standard deviation (n = 5).

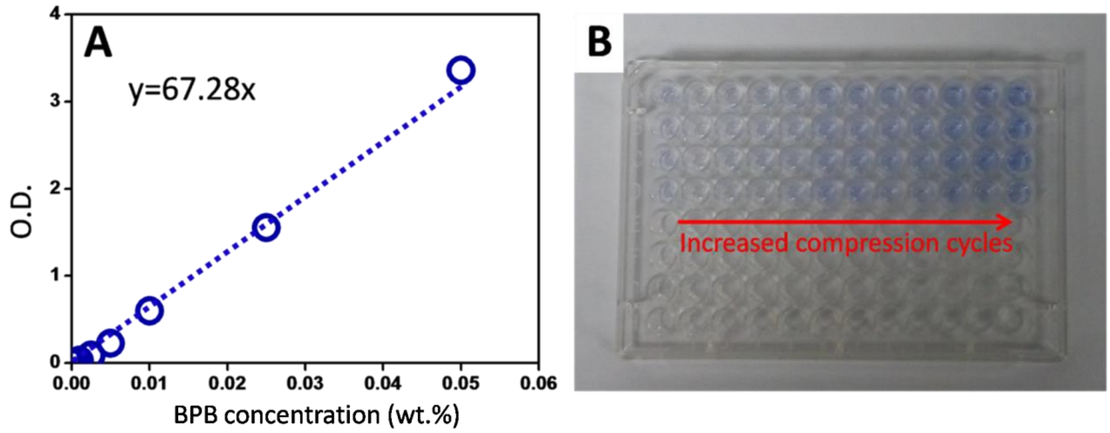

Figure S4. (A) Standard curve of the OD value vs. BPB solution with different concentration in 96-well plate; (B) BPB solution after increasing compression cycles.

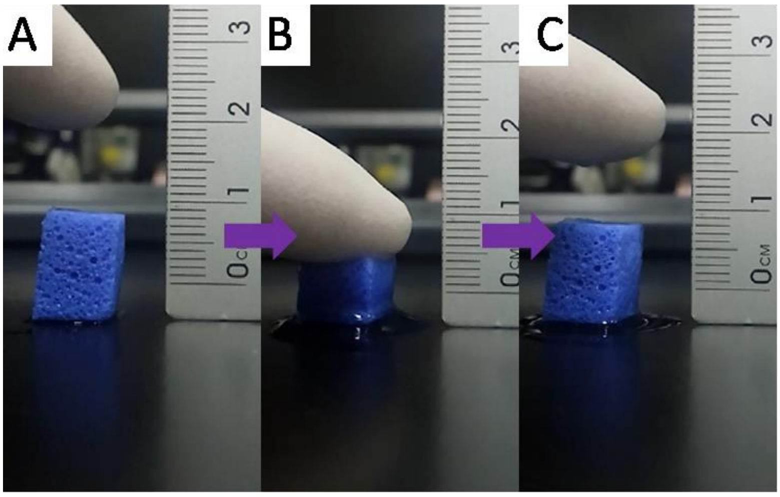

Movie S1. Resilience of the BPB-loaded CCS with 60 wt.% moisture content. CCS with appropriate moisture content exhibits high elasticity under the strain of < 50%.

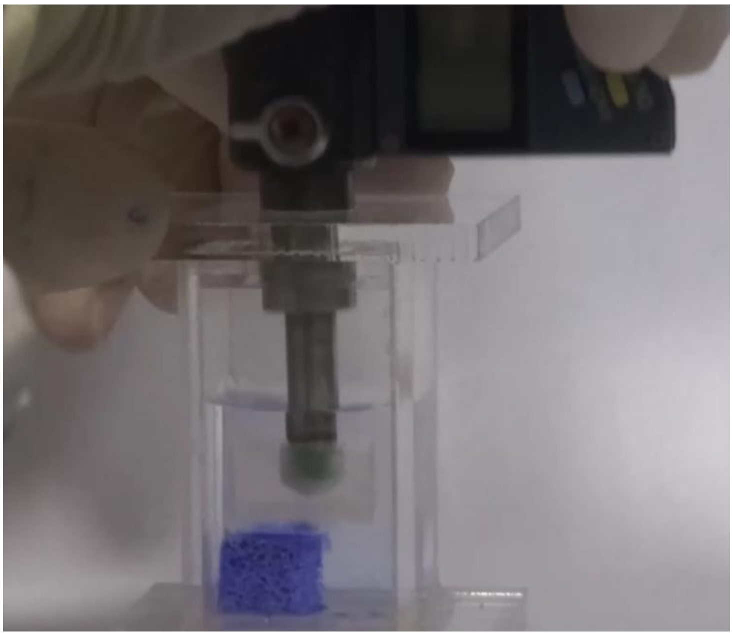

Movie S2. BPB released by cyclic compression from CCS with > 60% moisture content.

Table S1. The average amount of BSA released from unit volume of CCS after 150 compression cycles.

Compression set	1	2	3	4	5	15
BSA amount (ng/cm^3)	124±20	225±17	166±29	174±22	145±16	110±14

Table S2. BPB and fibroblast release from CCS compared with the release behavior of different cargoes from ferrogel (*)

Types of cargoes	BPB	Fibroblasts	Mitoxantrone* in ref [1]*	DNA* in ref [1]*	Fibroblasts (10% RGD used) in ref [1]*
Loading concentration (µg/mL or cells/mL carrier)	700	500,000	150,000	300	1,500,000
Amount of cargoes released from per mL carrier after one stimulus (ng/mL or cells/mL)	70	1400	113,000	44	5600
Percentage of cargoes released after one cycle (%)	0.01	0.28	0.075	0.015	0.38

*Data from the literature.[1]
**We did not use additional linkers or adhesive proteins to tether the hydrophobic BPB and cells.

Experimental Section

Materials: Food-grade cornstarch was purchased from Weimeisi Co., Ltd (Shanghai, China). Hydroxyapatite (HA) was synthesized in house according to a method described elsewhere.[2] Triton X-100 was purchased from Sinopharm Chemical Reagent Co., Ltd (Shanghai, China). Bromophenol blue (BPB) was procured from Sigma (St. Louis, MO).

Fabrication of CCS: Cornstarch and DI water was homogeneously mixed in a beaker to obtain 10 wt% starch suspension. Different amounts of HA powders were then added into the starch suspension and mixed uniformly to form ceramic slurries. For different purposes, the solid contents of HA (the ratio of HA mass to total solid mass) varied from 71.4% to 85.9%. The slurry was later heated to 90°C in a water bath and a surfactant of Triton X-100 was added. The heated suspension became highly viscous gel and then was vigorously stirred by an overhead stirrer till air bubbles fully infiltrated into the suspension to obtain foam. The foam was removed from water bath, cooled down to room temperature and then set for 24 hrs. The foam was further dried to porous composite scaffolds with wanted moisture contents, depending on different applications.

Fabrication of BPB- and BSA-loaded CCS: 8 g starch, different amounts of hydroxyapatite and appropriate volume of surfactant were mixed with 72 mL 0.1 wt.% BPB solution in a 200 mL beaker to obtain the uniform slurry, and then the slurry was heated to 90°C in a water bath to allow starch gelatinize. After that the slurry was foamed and set for 24 hrs to get the stable porous network. For BSA loading, the BPB solution was replaced by DI water. After foaming at 90°C, the beaker was placed in room temperature until the temperature of the slurry dropping to 50°C, 3.5 g of BSA (Sigma, St. Louis, MO) was added in the beaker and the slurry was foamed. The foam was set for 24 hrs to get the stable porous network. BPB- and BSA-loaded CCS could be cut to different shapes and dried by dehydration (dried by gradient

of ethanol solution) or freeze drying (-54°C) depending on different applications. BPB-loaded CCS could be cut to different shapes and dried by dehydration (dried by gradient of ethanol solution) or freeze drying (-54°C) depending on the applications.

Fabrication of porous HA ceramic by sintering CCS: Porous HA ceramics were sintered from dried CCS with 80% ceramic content. CCSs with the dimension of 15×15×15 mm^3 were sintered at 1500°C using muffle furnace (SJKQ-1700, Dingan Tec, Suzhou) for 2 hrs.

Characterization of CCS: Microstructure and morphology of CCS were characterized by scanning electron microscopy (SEM, FEI Quanta 250, acceleration voltage of 1.5 kV under a vacuum of 1.56×10^{-4} Pa). The sample was fractured and the facture surface for observation was sputtering-coated with Au-Pd. Dried CCSs with the dimension of 10×10×10 mm were scanned at the speed of 0.7°/s by Micro-CT (Skyscan1176) with the precision of 8.8 μm, and then three-dimensional microstructure of CCSs were obtained by reconstructions of micro-CT data.

Measurements of apparent density and porosity: Apparent density (ρ_{app}) of CCS and porous HA ceramic was calculated by its weight and volume that can be directly measured. Theoretical density (ρ_{th}) of the porous composites was calculated by the densities of the HA and starch according to their proportions in the composites. The porosity (p) of CCS was thus calculated by

$$p = \left(1 - \frac{\rho_{app}}{\rho_{th}}\right) \times 100\% \quad (1)$$

Mechanical characterization: For uniaxial compression tests, CCS were cut into cubes with dimension of 10×10×10 mm^3 and tested on a mechanical tester (HY-1080, testing range

0~500 N with precision of 0.01 N, Hengyi company, Shanghai) operating at a crosshead speed of 1mm/min. From the stress-strain curve, the maximum stress before failure was determined as compressive strength and the linear range in the stress curve before failure was used to calculate compressive modulus. For the testing of the compressive loop of CCS, CCSs with 45% moisture content were cut into cubes with dimension of 10×10×10 mm^3 and the cubes were pressed to desired strain of CCS (1%, 3%, 5% and 10%) and then unloaded to initial position. Resilience was characterized using a CCS sample with 45% water contents using microscope. A CCS cube was pressed to designated strains (3% and 10%), and released, and this process was video-recorded by microscope. The resilience was then calculated by comparing the position of rebounding surface to its initial position.

BPB Release behavior of CCS: Release behavior of CCS loaded with BPB was studied by compressing CCS cubes (10×10×10 mm^3) at different strains (0.1%, 0.2%, 0.5%, 0.8%, 1%, 2%, 3%, 5%, 6%, and 8%) in a series of ethanol/water solution (water contents 0, 20%, 40%, 60%, 80% and 100%). After pressing for 10 times, the solution containing released BPB was retrieved and spectrophotometrically measured on a microplate reader (at 450 nm on BioTek MQX200R). Optical densities (OD) of the solution were compared to a standard OD curve of solution containing known concentrations of BPB (see SI and Figure S4 for more information). Meanwhile, a CCS cube that was placed in the same ethanol/water solution but not pressed was used as a control to calculate the BPB release from CCS without mechanical stimulation. For cyclic release tests, CCS was compressed at designated strains for 50 consecutive cycles (defined as one set of compression) and the solution containing released BPB was removed for spectrophotometical measurement. Then test resumed for another set and repeated up to 6 sets in total. A parallel test of the CCS without compression was used to determine the background release. The amount of BPB released in each set subtracted the background release was averaged by 50 cycles and then total volume of CCS to obtain the net

release amount per cycle per cm^3 of CCS. All the releases tests were repeated at least three times.

Calculation of the amount of BPB released from CCS: 100 μL of BPB solution with different concentration was placed in a 96-well plate, and then the solution was tested by spectrophotometrically measured on a microplate reader at a wavelength of 450 nm (BioTek MQX200R). The as-measured OD value was corrected by subtracting the background of DI water. After that, the standard curve of BPB concentrations vs. OD values was obtained. The experiments were repeated for at least three times. In order to calculate the amounts of the BPB released from the porous composite scaffolds with different compressive strains, the as-measured OD value was corrected by subtracting the background of DI water. The concentration of the BPB solution (c) could be calculated by comparing with the standard curve. Assuming the total volume of the solution BPB released in and the volume of the CCS cubes were v_1 and v_2, respectively. Then the amount of BPB released from per cm^3 CCS could be calculated as:

$$m = \frac{\varepsilon v_1}{v_2} \qquad (2)$$

Measurement of the amount of BSA released from CCS: For cyclic release of BSA test, BSA-loaded CCS was compressed at designated strains (3%) for 10 consecutive cycles (defined as one set of compression) and the solution containing released BAS was removed for spectrophotometical measurement (100 μL to 96-well plate, 570 nm) before treated with Micro BCATM Protein Assay Kit (Prod#23235, Thermo Scientific). Then test resumed for another set and repeated up to 15 sets in total. A parallel test of the CCS without compression was used to determine the background release. The amount of BSA was measured depend on the instruction of the protein kit, and then the amount of BSA released in each set subtracted the background release was averaged by 10 cycles and then total volume of CCS to obtain the

net release amount per cycle per cm^3 of CCS. All the releases tests were repeated at least three times.

Cell release test: Cell-loaded CCS was immersed in cell culture medium (89% αMEM, 10% FBS and 1% P/S) and then subjected to cyclic compressions at a strain of 20%. CCS was compressed for 20 consecutive cycles (defined as one set of compression) and the cell culture medium containing released cells was removed for a 24-hr culture and then cell count by the LIVE/DEAD® Viability/Cytotoxicity Kit (Thermo Fisher Scientific, L-3224) according to its instructions. Then test resumed for another set and repeated up to 4 sets in total. A parallel test of the CCS without compression was used to determine the background release of cells. Live/dead cells after staining were imaged by fluorescence microscopy (ZEISS, AxioCamHRc) and the number of live cells was counted. The number of live cells released in each set subtracted the number of background release and was averaged by 20 cycles and then total volume of CCS to obtain the net released cell number per cycle per cm^3 of CCS. All the releases tests were repeated three times.

Theoretical estimation of ΔP *and* ε_{repel}*:* In a simplified, one-end closed cylindrical pore model, the hydrophilic liquid is drawn into the pores due to capillary effect and the gas pressure in the pore is described as:

$$P_0 = P - \Delta P \quad (3)$$

Where P is the atmosphere pressure, and ΔP is capillary pressure given by Young-Laplace equation:

$$\Delta P = \frac{2\gamma\cos\theta}{r} \quad (4)$$

Where γ is the surface tension of hydrophilic liquid, θ the contact angle between water and material of cylindrical pore, and r is the radius of the cross section of pore.

Assuming change of gas pressure is due to the volume change of the pore and the gas obeys ideal gas law:

$$PV = nRT \tag{5}$$

Where n is moles of gas, R ideal gas constant, and T is temperature.

Assume during volume contraction the cross section of the cylinder becomes an ellipse, and the initial and changed gas pressures (P_0 and P_1, respectively) have a relationship given by ideal gas law:

$$\frac{P_0}{P_1} = \frac{V_1}{V_0} = \frac{ab}{r^2} \tag{6}$$

Where $V_1$and V_2 are original and compressed volume of gas, respectively, and a and b are short and long axis of the cross-sectional ellipse, respectively.

In order to expel the liquid out of the pore, gas pressure P_1 at least needs to be equal to P, so the equation (6) rewrites as:

$$\frac{P-\Delta P}{P} = \frac{ab}{r^2} = 1 - \frac{2\gamma\cos\theta}{Pr} \tag{7}$$

Considering the poisson's ratio ν of CCS:

$$b = \frac{r-a}{r} \times \nu + r \tag{8}$$

Solve equations (7) and (8) to obtain:

$$a = \frac{r^2+r\nu-\sqrt{(r^2+\nu r)^2-4\nu r^3(1-\frac{2\gamma\cos\theta}{Pr})}}{2\nu}, (a<r) \tag{9}$$

When expelling the liquid by compressing CCS, the expelling strain is thus given by,

$$\varepsilon \geq \left(1-\frac{a}{r}\right) \times 100\% = \left(1-\frac{r^2+r\nu-\sqrt{(r^2+\nu r)^2-4\nu r^3(1-\frac{2\gamma\cos\theta}{Pr})}}{2\nu r}\right) \times 100\% \tag{10}$$

For calculation, poisson's ratio of 0.2 is measured from experiments, and surface tension of water under the experimental temperature is 7.2 $\times 10^{-2}$ N/m, and the contact angle of HA was assumed equal to 10°.

[1] X. Zhao, J. Kim, C. A. Cezar, N. Huebsch, K. Lee, K. H. Bouhadir, D. J. Mooney, *PNAS* **2011**, *108*, 67.

[2] A. Ebrahimpour, M. Johnsson, C. F. Richardson, G. H. Nancollas, *J. Colloid Interface Sci.* **1993**, *159*, 158.
